# A Geometry-Based Cycle Slip Detection and Repair Method with Time-Differenced Carrier Phase (TDCP) for a Single Frequency Global Position System (GPS) + BeiDou Navigation Satellite System (BDS) Receiver

**DOI:** 10.3390/s16122064

**Published:** 2016-12-05

**Authors:** Chuang Qian, Hui Liu, Ming Zhang, Bao Shu, Longwei Xu, Rufei Zhang

**Affiliations:** GNSS Research Centre, Wuhan University, No. 129 Luoyu Road, Wuhan 430079, China; qc_gnss@whu.edu.cn (C.Q.); mingz@whu.edu.cn (M.Z.); baos613@whu.edu.cn (B.S.); lw_xu@whu.edu.cn (L.X.); zhangrufeihf@163.com (R.Z.)

**Keywords:** GPS + BDS, TDCP, single frequency, cycle slip, ILAM, outlier

## Abstract

As the field of high-precision applications based on carriers continues to expand, the development of low-cost, small, modular receivers and their application in diverse scenarios and situations with complex data quality has increased the requirements of carrier-phase data preprocessing. A new geometry-based cycle slip detection and repair method based on Global Position System (GPS) + BeiDou Navigation Satellite System (BDS) is proposed. The method uses a Time-differenced Carrier Phase (TDCP) model, which eliminates the Inner-System Bias (ISB) between GPS and BDS, and it is conducive to the effective combination of GPS and BDS. It avoids the interference of the noise of the pseudo-range with cycle slip detection, while the cycle slips are preserved as integers. This method does not limit the receiver frequency number, and it is applicable to single-frequency data. The process is divided into two steps to detect and repair cycle slip. The first step is cycle slip detection, using the Improved Local Analysis Method (ILAM) to find satellites that have cycle slips; The second step is to repair the cycle slips, including estimating the float solution of changes in ambiguities at the satellites that have cycle slips with the least squares method and the integer solution of the cycle slips by rounding. In the process of rounding, in addition to the success probability, a decimal test is carried out to validate the result. Finally, experiments with filed test data are carried out to prove the effectiveness of this method. The results show that the detectable cycle slips number with GPS + BDS is much greater than that with GPS. The method can also detect the non-integer outliers while fixing the cycle slip. The maximum decimal bias in repair is less than that with GPS. It implies that this method takes full advantages of multi-system.

## 1. Introduction

Precise navigation and positioning with the Global Navigation Satellite System (GNSS) depends on high-precision carrier phase observations, which require accurate estimations of ambiguity. The causes of cycle slip include blocked signals, low signal-to-noise ratios (SNR), and errors in signal processing. They will interrupt the continuous tracking of carrier phase observations, result in the occurrence of cycle slips and even outliers in severe cases. The impact of cycle slips must be considered when obtaining the ambiguity [1]. Cycle slip detection and repair can avoid re-determining the ambiguity and improve the continuity of positioning, which has been studied for many years.

Existing methods for fixing cycle slip methods are primarily divided into two types. The first one treats each satellite separately, using combinations of observations to detect cycle slips. For example, some of them use double difference observations [2,3,4,5] to detect cycle slips, which are applied in multi-station situations. And they are not suitable for a single station; The methods based on Global Position System (GPS) data and other sensor observations [5,6,7] require supplementary information from the inertial navigation system (INS), which significantly constrains their feasibility in many applications, due to the cost and complexity of adding an INS system to GPS [8]; Methods based on combinations of phase, code and Doppler observations [8,9,10,11,12] are the main cycle slip fixing methods for single station, which have their own shortcomings: Methods based on combinations of carrier phase observations are supported by multiple frequencies, they are not available for single frequency; Methods based on combining observations with pseudo-range is limited by the noise level. Doppler is the instantaneous shift in the measured frequency and suitable to detect and correct cycle-slips. But the deviation in the receiver’s oscillator clock may result in Doppler measurement error [13]. The methods treat each satellite separately, thereby cannot take advantage of the current multi-constellation.

The second type of method is geometry-based, which treat the satellite constellation as a whole. Most of these methods utilizes the time-differenced model. Zhang and Li [14] predicted the atmospheric delays and fixed cycle slip in dual-frequency PPP with a time-differenced model, and Ye et al. [15] expanded the method to GPS + Global Navigation Satellite System (GLONASS) observations. Carcanague [16] utilized the time-differenced model to fix cycle slip for single frequency GPS + GLONASS observations. These methods utilize pseudo-range or Doppler observations to calculate a float solution of time-differenced changes in the ambiguity, searching for integer solutions with the least-squares ambiguity decorrelation adjustment (LAMBDA) to obtain the integer cycle slips. Some methods utilize outlier detection, such as quasi-accurate detection of outliers (QUAD) [17], the statistical hypothesis tests [18,19], robust estimation with single frequency data [20] and so on, which adopt the residuals after the least squares adjustment to detect cycle slips and a rounding algorithm to obtain the integer cycle slips.

Geometry-based methods are the current trend in multi-constellation development and more flexible and suitable for single-frequency receivers, but most of the existing geometry-based methods do not take the non-integer outliers into account. When non-integer outliers exist, the integer search with LAMBDA may fail, and the rounding algorithm have not applied validation process to detect them. In addition, the methods based on LAMBDA are influenced by the precision of the pseudo-range or Doppler observations. Moreover, the methods focused on GPS and GLONASS, while little has combined BeiDou Navigation Satellite System (BDS).

As the preceding review shows, the time-differenced carrier phase (TDCP) technique is selected to combine the single frequency GPS + BDS for fixing cycle slips, which consists in differencing successive carrier phases. It can eliminate the constant integer ambiguities, inner-system bias (ISB) and most of the common mode errors, which vary slowly within a short period of time like 1 s [21], and obtain precise position change at the millimeter level [22]. Compared to existing geometry-based methods, this paper offers several major contributions: (1) improved local analysis method (ILAM) is proposed and applied in time-differenced carrier phase (TDCP) algorithm to detect cycle slip. The method can effectively detect the number of cycle slips and the corresponding satellites. As the increase of the number of satellites in multi-constellation, detectable cycle slips number is more; (2) success probability and decimal test are utilized for cycle slips validation. The non-integer outlies can be detected, which avoids the error repairs and improves the reliability. The experiment based on GPS + BDS proves the effectiveness and the advantages in multi-constellation.

The following section first describes the TDCP algorithm and then gives a detailed description of cycle slips detection with ILAM and repair by rounding with validation. In the subsequent section, the proposed method is verified with real receiver data and simulated cycle slips, and the conclusions follow.

## 2. Methodology

The principle of detection and repair is given in this section. The TDCP model based on GPS and BDS observations is introduced and analyzed first. Thereafter, we present the detection method with ILAM. Finally, we describe the validation process during repair.

### 2.1. TDCP Model

GPS and BDS carrier phase measurements on frequency L1/B1 can be expressed as follows:

(1) $$\lambda^{j}\varphi^{j}=\rho^{j}+c(t_{G}-t^{j})-I^{j}+T^{j}+\lambda^{j}N^{j}+\varepsilon^{j}$$

(2) $$\lambda^{k}\varphi^{k}=\rho^{k}+c(t_{C}-t^{k})-I^{k}+T^{k}+\lambda^{k}N^{k}+\varepsilon^{k}$$

where the superscript *j* stands for a GPS satellite and *k* for a BDS satellite, φ is the carrier phase measurement, is the carrier phase wavelength, λ is the geometric range, *c* is the speed of light in vacuum, $t_{G}$ and $t_{C}$ are the receiver clock biases for GPS and BDS, respectively, while $t^{j}$ and $t^{k}$ are the satellite clock biases, *I* and *T* are the slant ionospheric and tropospheric delay, respectively, *N* is the integer ambiguity, and ε includes multipath and receiver noise.

The BDS receiver clock bias can be expressed as the sum of the GPS receiver clock bias and the inner-system bias (ISB) between GPS and BDS; thus:

(3) $$t_{C}=t_{G}+{ISB}_{C-G}$$

where $ISB_{C-G}$ is the ISB between GPS and BDS defined by their clock products. Substituting Equation (3) into Equation (2) gives:

(4) $$\lambda^{k}\varphi^{k}=\rho^{k}+c(t_{G}+ISB_{C-G}-t^{k})-I^{k}+T^{k}+\lambda^{k}N^{k}+\varepsilon^{k}$$

The TDCP method adopts the single differences in time of the one-way carrier phase measurements. The TDCP measurements are the time differences of successive carrier phases to the same satellite. According to Equations (1) and (4), the difference between carrier phase measurements at two successive epochs $t_{m}$ and $t_{m-1}$ is:

(5) $$\lambda^{j}\Delta \varphi_{t_{m-1},t_{m}}^{j}=\lambda^{j}(\Delta \varphi_{t_{m}}^{j}-\Delta \varphi_{t_{m-1}}^{j})=\Delta \rho^{j}+c(\Delta t_{G}-\Delta t^{j})-\Delta I^{j}+\Delta T^{j}+\Delta \varepsilon^{j}$$

(6) $$\lambda^{k}\Delta \varphi_{t_{m-1},t_{m}}^{k}=\lambda^{k}(\Delta \varphi_{t_{m}}^{k}-\Delta \varphi_{t_{m-1}}^{k})=\Delta \rho^{k}+c(\Delta t_{G}+\Delta ISB_{C-G}-\Delta t^{k})-\Delta I^{k}+\Delta T^{k}+\Delta \varepsilon^{k}$$

where Δ represents the differencing operation. For example, $\Delta \rho^{j}=\rho_{t_{m}}^{j}-\rho_{t_{m-1}}^{j}$ is the change in the geometric range between two epochs, and the other terms in Equations (5) and (6) are defined accordingly. The integer ambiguity is not included in Equations (5) and (6) as long as a cycle slip does not occur. To linearize Equations (5) and (6), the individual carrier phase measurements have to be corrected. Corrections for satellite clock errors are extracted from ephemeris data, while the ionospheric and tropospheric models are used to mitigate the influence of ionospheric and tropospheric effects, respectively. Therefore, satellite clock bias, ephemeris errors and slant ionospheric and tropospheric delay terms become time differences of residual errors and are negligible [21].

Therefore, the compensated TDCP measurement is:

(7) $$\lambda^{j}\Delta {\tilde{\varphi }}_{t_{m-1},t_{m}}^{j}=\Delta \rho^{j}+c\Delta t_{G}+\Delta \varepsilon^{j}$$

(8) $$\lambda^{k}\Delta {\tilde{\varphi }}_{t_{m-1},t_{m}}^{k}=\Delta \rho^{k}+c(\Delta t_{G}+\Delta ISB_{C-G})+\Delta \varepsilon^{k}$$

**Figure 1:**
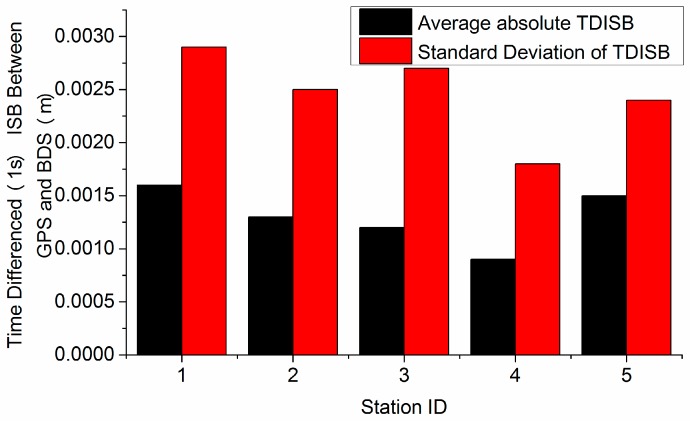
Statistics of time-differenced inter-system bias (TDISB) for five sites on DOY 353 in 2015.

ISBs consist of the difference in time scales between two constellations and the difference in hardware delays, and are quite stable [23]. ISBs have been applied to ensure the compatibility and interoperability of multi-GNSS [24]. The 1-s interval GPS and BDS receiver clock biases of 5 sites on day of the year (DOY) 353 in 2015 were calculated with back-smoothed precise point positioning (PPP). The ISBs for each site were then calculated by differencing the GPS and BDS receiver clock biases. Furthermore, the 1-s interval ISB values are differenced between successive epochs and converted to the related distance. These statistics are shown in Figure 1.

Figure 1 shows that the 1-s interval TDISB has an average absolute value of 2 mm with an STD of 3 mm for each station. This means that we can obtain the estimate of the TDISB with very high precision. Therefore, the ISB is rather stable within 1 s and can be eliminated by TDCP. This implies that only one parameter of receiver clock variation is needed to combine GPS and BDS. Thus, the equation for both systems can be written as follows:

(9) $$\lambda \Delta \tilde{\varphi }=\Delta \rho +c\Delta t+\Delta \varepsilon$$

where Δε includes the errors relative to multipath and receiver noise and the residuals of the partially corrected error sources. After linearization, Equation (9) becomes:

(10) $$\lambda \Delta \tilde{\varphi }=\left[e^{x}\;e^{y}\;e^{z}\;1\right]\cdot \left[\begin{array}{c} \Delta r_{u}^{x}\\ \Delta r_{u}^{y}\\ \Delta r_{u}^{z}\\ c\Delta t \end{array}\right]+\Delta \varepsilon$$

where $e^{x}$, $e^{y}$ and $e^{z}$ are line-of-sight three-dimensional components, and $\Delta r_{u}^{x}$, $\Delta r_{u}^{y}$ and $\Delta r_{u}^{z}$ are receiver position change three-dimensional components.

### 2.2. Cycle Slip Detection with ILAM

There are many methods for eliminating or reducing the impact of outliers. In these methods, robust estimation methods adjust the stochastic model to reduce the impact of outliers on the results; other methods are based on statistical principles [25], and have been extended to multiple outliers [26] and applied [27,28]. However, these methods are based on residuals of adjustment, while the residuals of observations with outliers may be not larger [29]. To detect multiple outliers is still an open problem [26].

The local analysis method (LAM) is proposed to address the shortcomings of the existing outliers detecting methods based on residuals, and it does not depend on residuals of adjustment. The core idea is that an observed quantity can be expressed as combinations of other observed quantities, and the observed quantity and the combinations can be compared to detect outliers [29]. This method assumes that there are no outliers in the observed quantity or in the other observed quantities included in the combinations if the observed quantity and combinations meet certain conditions. This method does not consider the possibility that outliers may exactly offset each other, which would lead to incorrect detection. We improve the LAM to detect cycle slip from the statistical point of view by considering all of the combinations, rather than a single combination, called ILAM.

According to LAM, the observed quantity of satellite *i* can be written as a combination of 4 other observed quantities of satellites 1, 2, 3 and 4:

(11) $$\lambda \Delta {\tilde{\varphi }}_{i}=a_{1}\lambda \Delta {\tilde{\varphi }}_{1}+a_{2}\lambda \Delta {\tilde{\varphi }}_{2}+a_{3}\lambda \Delta {\tilde{\varphi }}_{3}+a_{4}\lambda \Delta {\tilde{\varphi }}_{4}$$

where $\left[a_{1}\;a_{2}\;a_{3}\;a_{4}\right]$ need to be determined.

According to Equation (10), the observed quantity of satellite *i* can be expressed:

(12) $$\lambda \Delta {\tilde{\varphi }}_{i}=B_{i}\cdot X$$

where:

$$B_{i}=\left[e_{i}^{x}\;e_{i}^{y}\;e_{i}^{z}\;1\right]\;X=\left[\begin{array}{c} \Delta r_{u}^{x}\\ \Delta r_{u}^{y}\\ \Delta r_{u}^{z}\\ c\Delta t \end{array}\right]$$

Similarly, the four observed quantities of satellites 1, 2, 3 and 4 can be expressed by:

(13) $$L=B_{4}\cdot X$$

where:

$$L=\left[\begin{array}{c} \lambda \Delta {\tilde{\varphi }}_{1}\\ \lambda \Delta {\tilde{\varphi }}_{2}\\ \lambda \Delta {\tilde{\varphi }}_{3}\\ \lambda \Delta {\tilde{\varphi }}_{4} \end{array}\right]\;B_{4}=\left[\begin{array}{c} e_{1}^{x}\;e_{1}^{y}\;e_{1}^{z}\;1\\ e_{2}^{x}\;e_{2}^{y}\;e_{2}^{z}\;1\\ e_{3}^{x}\;e_{3}^{y}\;e_{3}^{z}\;1\\ e_{4}^{x}\;e_{4}^{y}\;e_{4}^{z}\;1 \end{array}\right]$$

If $B_{4}$ is invertible and Equation (13) is converted to:

(14) $$X=B_{4}^{-1}\cdot L$$

Substituting Equation (14) into Equation (12) gives

(15) $$\lambda \Delta {\tilde{\varphi }}_{i}=B_{i}\cdot B_{4}^{-1}\cdot L$$

Combining Equations (11) and (15) gives:

(16) $$[a_{1}\;a_{2}\;a_{3}\;a_{4}]=B_{i}\cdot B_{4}^{-1}$$

assuming:

(17) $$w=\lambda \Delta {\tilde{\varphi }}_{i}-(a_{1}\lambda \Delta {\tilde{\varphi }}_{1}+a_{2}\lambda \Delta {\tilde{\varphi }}_{2}+a_{3}\lambda \Delta {\tilde{\varphi }}_{3}+a_{4}\lambda \Delta {\tilde{\varphi }}_{4})$$

Equation (11) reveals that the true value of w is small. The true error of w is computed by Equation (17). According to the law of error propagation, the error of mean squares $\sigma_{w}$ of w can be calculated based on the precision of the TDCP observations. In the experiment, we assume the precision of raw TDCP is 5 mm including the errors relative to multipath, receiver noise, and the residuals of the partially corrected error sources. This value is related with receivers and troposphere and ionosphere environment. Additionally, the elevation-dependent measurement weighting method is considered.

When $\left|w\right|\leq 2\sigma_{w}$ (assuming random errors are normally distributed), the combination of these four satellites can be treated as a repeat observation of satellite *i*. Except for satellite *i*, any combination of four satellites can constitute a combination of satellite *i*. Any satellite has the same number of combinations as an observed quantity. If the numbers of combinations and repeat observations of one observed quantity are k and m, respectively, the repetitive rate $P_{A}$ is defined as:

(18) $$P_{A}=\frac{m}{k}$$

Theoretically, there is no repeat observation in the combinations if cycle slips exist in the observed quantity of one satellite. The number of repeat observations in the combinations is determined by the number of satellites and cycle slips if there is no cycle slip in the observed quantity of one satellite. Therefore, we can get the theoretical value of the repetitive rate by:

(19) $$P_{T}=\{\begin{array}{cc} \frac{C_{n-t-1}^{4}}{C_{n-1}^{4}} & \left(\text{there is no cycle slip}\right)\\ 0 & \left(\text{there is cycle slip}\right) \end{array}$$

where *n* and *t* are the number of satellites and cycle slips, respectively. We can determine the number of cycle slips based on the repetition rates of the satellites that have no cycle slips and detect the satellites that have cycle slips based on the repetitive rates. Because of the influence of random factors, $P_{A}$ should be close to $P_{T}$, but not equal.

**Figure 2:**
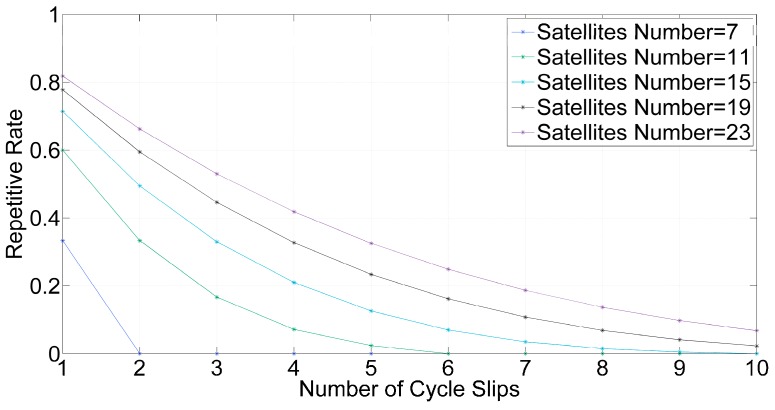
Repetitive Rate under Different Numbers of Satellites and Cycle Slips.

$P_{T}$ increases as the number of satellites increases and decreases as the number of cycle slips increases. Figure 2 shows the $P_{T}$ changes under incremental numbers of satellites and cycle slips.

The detection process can be divided into two steps. The first step is to search the number of cycle slips *t*, and the second step is to determine which satellites have cycle slips. Each satellite will be calculated in all combinations to obtain the repetitive rate. The repetitive rates of all of the satellites will be sorted by descending order. Hypothetically, the numbers of satellites and cycle slips are *n* and *t*, respectively, and the number of valid satellites is *n* − *t*. Because the repetition rates of valid satellites would certainly be larger than those of abnormal satellites, which are close to zero, the larger repetition rates for *n* − *t* correspond to the valid satellites. Considering the theoretical value $P_{T}$ corresponding to *t* cycle slips, the standard deviation can be calculated by:

(20) $$\sigma =\sqrt{\frac{1}{n-t}\sum_{i=1}^{n-t}{\left(P_{A}-P_{T}\right)}^{2}}$$

**Figure 3:**
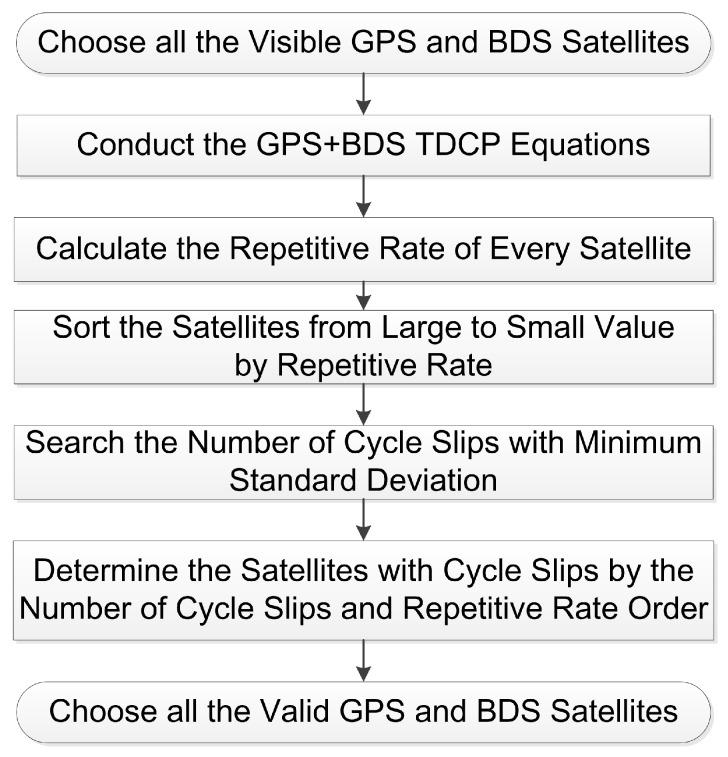
Cycle Slips Detection Flow of ILAM.

We can calculate the standard deviations of different *t* and find the minimum value that corresponds to the correct number of cycle slips. After the number of cycle slips t is known, the *t* smaller repetition rates correspond to the cycle slips. This specific process is shown in Figure 3.

### 2.3. Cycle Slip Repair Method with Success Probability

Hypothetically, there are *m* satellites and *t* cycle slips. After we use the ILAM to determine which satellites have cycle slips, it is possible to make use of the least square to estimate the position parameters, clock error parameters and cycle slips parameters:

(21) $$X={\left(B^{T}\cdot P\cdot B\right)}^{-1}\cdot B^{T}\cdot P\cdot l$$

where:

$$X=\left[\begin{array}{c} \Delta r_{u}^{x}\\ \Delta r_{u}^{y}\\ \Delta r_{u}^{z}\\ c\Delta t\\ \Delta N_{1}\\ \vdots \\ \Delta N_{t} \end{array}\right]\;B=\left[\begin{array}{ccccccc} e_{1}^{x} & e_{1}^{y} & e_{1}^{z} & 1 & \lambda_{1} & & \\ \vdots & \vdots & \vdots & \vdots & & \ddots & \\ e_{t}^{x} & e_{t}^{y} & e_{t}^{z} & 1 & & & \lambda_{t}\\ \vdots & \vdots & \vdots & \vdots & & & \\ e_{m}^{x} & e_{m}^{y} & e_{m}^{z} & 1 & & & \end{array}\right]\;l=\left[\begin{array}{c} \lambda_{1}\Delta {\tilde{\varphi }}_{1}\\ \lambda_{2}\Delta {\tilde{\varphi }}_{2}\\ \vdots \\ \lambda_{m}\Delta {\tilde{\varphi }}_{m} \end{array}\right]$$

The values of matrix *P* are calculated by the elevation-dependent measurement weighting method.

After calculating a float solution, searching for integer cycle slips is the next step to repair the cycle slips. The LAMBDA method is a popular and effective method to search integer ambiguity [30], when the parameters’ true values are all integers. But in reality, the non-integer outliers may occur, and the integer search may fail. A separately rounding method is available to obtain integer cycle slips. The success probability is an ambiguity validation method for ambiguity rounding and is calculated as [31]:

(22) $$P\left(\Delta N=\Delta \overset{⌣}{N}\right)=P(\left|\Delta N-\Delta \hat{N}\right|\leq \frac{1}{2})=2\phi \left(\frac{1}{2\sigma_{\Delta \hat{N}}}\right)-1$$

where $\Delta \hat{N}$ is the float solution of ΔN, $\Delta \overset{⌣}{N}$ is the closest integer of ΔN, ϕ(x) meets the standard normal distribution, and $\sigma_{\Delta \hat{N}}$ is the standard deviation of $\Delta \hat{N}$.

Because the standard deviations of float solutions of cycle slips and outliers are similar by this method, the success probability cannot detect the non-integer outliers. The decimal test can solve this problem because the fractional part of the cycle slip is bound by the standard deviation. We can detect outliers with the formula:

(23) $$\left|\Delta \hat{N}-\Delta \overset{⌣}{\hat{N}}\right|\leq 3\sigma_{\Delta \hat{N}}$$

where $\Delta \overset{⌣}{\hat{N}}$ is the closest integer of $\Delta \hat{N}$. The float solution can be rounded and can repair cycle slips when Equation (23) is true. If the float solution does not meet Equation (23), there is outlier at this satellite.

## 3. Results and Discussion

To verify the proposed method, we collected data by TRIMBLE NETR9 and TRM57971.00 on the roof of the Wuhan University laboratory building from GPS time 11:35 to 14:20 10 April 2015. The sampling rate is 1 Hz, the cutoff elevation angle is set as 15°, and the frequencies are B1 for BDS and L1 for GPS. The raw data were carefully checked and confirmed to be cycle slip and outlier free, to simulate cycle slips accurately. In this section, we first examined the ILAM method to detect simulated cycle slips. We then tested the rounding method for cycle slip repair with success probability and decimal test as the validation method.

### 3.1. ILAM for Detecting Cycle Slips

**Figure 4:**
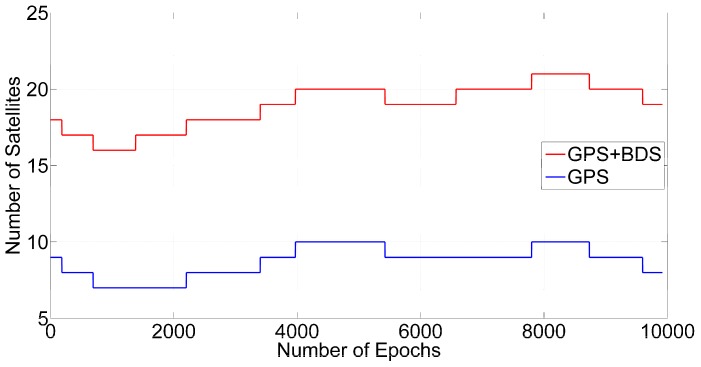
Satellite numbers for GPS and GPS + BDS.

The detection results of ILAM depend on the number of satellites and cycle slips. To facilitate a statistical analysis, we merged the periods with the same number of satellites together for analysis. Figure 4 shows the number of satellites in this period. There are 7–10 GPS satellites and 16–21 GPS + BDS satellites at the epochs.

**Figure 5:**
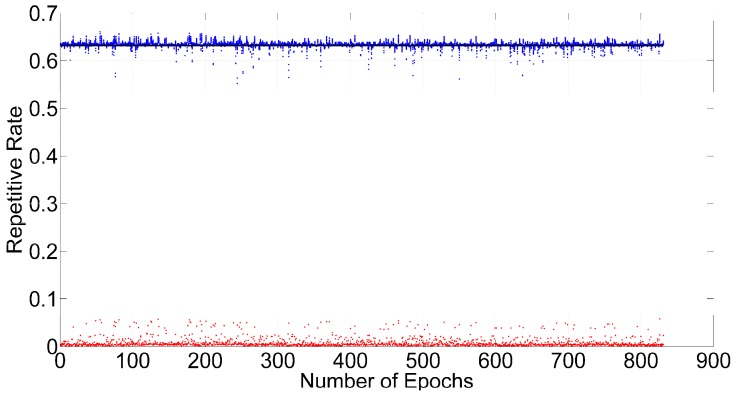
Repetitive rates of 21 satellites with two cycle slips (blue dots for valid satellites, red dots for satellites with cycle slips, and black line for theoretical value).

The epochs with 21 satellites were chosen to test ILAM. Two satellites are selected to add random cycle slips at every epoch. First, each satellite will be selected to obtain the repetitive rate. Figure 5 shows the repetitive rates of every satellite.

**Figure 6:**
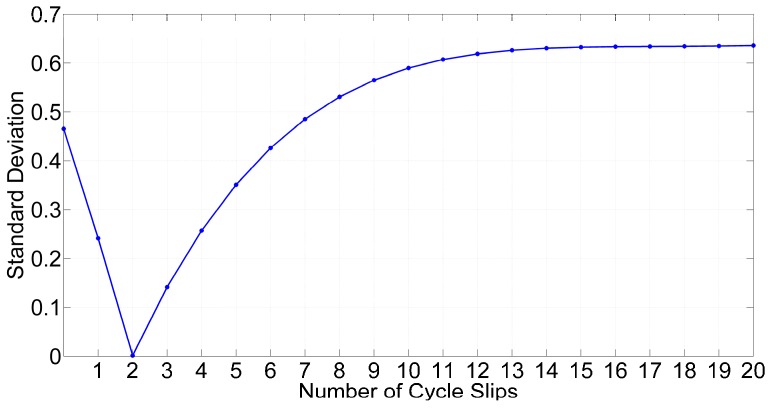
Standard deviations with different numbers of cycle slips at one epoch.

The results shown in Figure 5 demonstrate that the repetitive rates of valid satellites are near the theoretical value, while the repetitive rates of abnormal satellites are close to zero. We then use Equation (20) to calculate the standard deviation by different numbers of cycle slips. Figure 6 shows changes in the standard deviation by different assumptions for the numbers of cycle slips at one epoch. When the number of cycle slips is two, which is the correct value, the standard deviation reaches its minimum value. We can determine the number of cycle slips by the standard deviation method. The final step is determining the corresponding satellites with lower repetition rates.

To ensure the correct selection of the satellites with cycle slips, the maximum repetitive rate of abnormal satellites should be smaller than the minimum repetitive rate of valid satellites. The repetition rates of the valid satellites are much larger than those of the anomalous satellites, as shown in Figure 5, and cycle slips can be detected successfully when there are 21 satellites and two cycle slips. Therefore, two conditions must be met to detect cycle slips successfully with ILAM: one is that determining the correct number of cycle slips; the other one is, that the minimum repetitive rate of satellites without cycle slips is greater than the maximum repetitive rate of satellites with cycle slips. 

**Figure 7:**
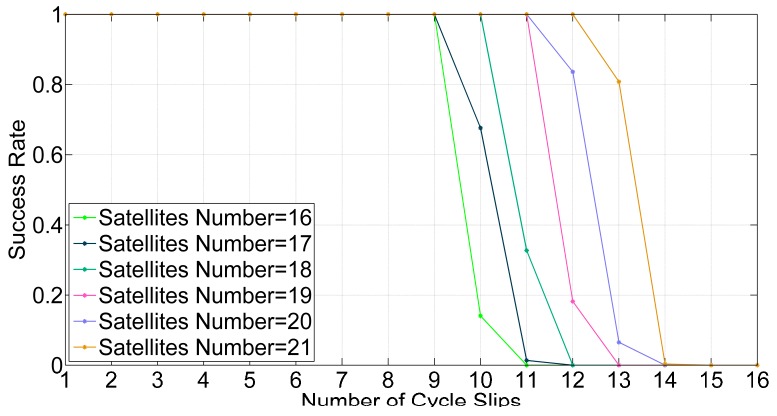
Success rates of detecting cycle slips by different numbers of satellites and cycle slips with GPS + BDS.

According to Equation (19), the repetition rates of valid satellites will decrease when there are more cycle slips, which may lead to a failure to detect cycle slips. The detectable cycle slips number is an effective indicator to test detection capacity. By putting two conditions as criteria, we can judge if cycle slips can be detected successfully in one epoch. The proportion of successful epochs represents the success rates of cycle slip detection. The success rates under the conditions of different numbers of satellites are calculated by adding different numbers of cycle slips to the GPS + BDS, as shown in Figure 7.

**Figure 8:**
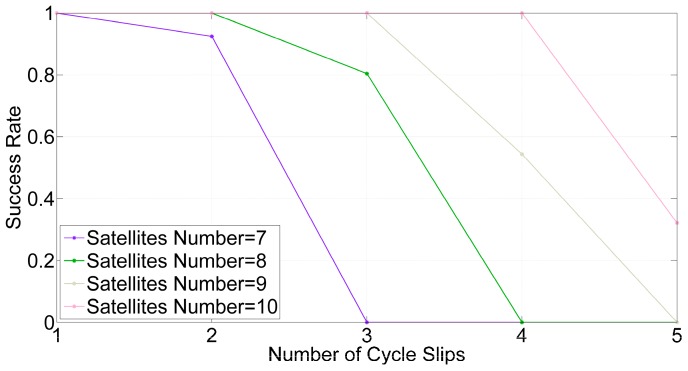
Success rates of detecting cycle slips by different numbers of satellites and cycle slips with GPS.

To compare the conditions of fewer satellites, the success rates with GPS are calculated by the same methods, as shown in Figure 8.

If 99% is set as the threshold value of the success rate to determine whether the cycle slips can be reliably detected, the detectable cycle slips numbers for different numbers of satellites are estimated from Figure 7 and Figure 8, and these results are listed in Table 1. Obviously, with GPS + BDS, there are more satellites and the detectable cycle slips number is more than that with GPS. The mean numbers satellites are 18 and eight, respectively with GPS + BDS and GPS. Compared to GPS, the mean detectable cycle slips number increases by four-fold, from 2 to 10 with GPS + BDS. Therefore, ILAM method can take advantage of the multi-constellation.

### 3.2. Success Probability and Decimal Test for Cycle Slip Repair

**Figure 9:**
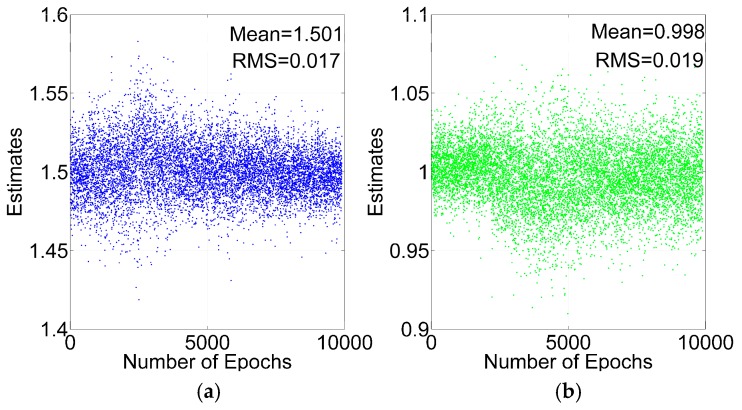
Cycle slips estimates for GPS + BDS: 1.5 Cycles (**a**); 1 Cycle (**b**).

**Figure 10:**
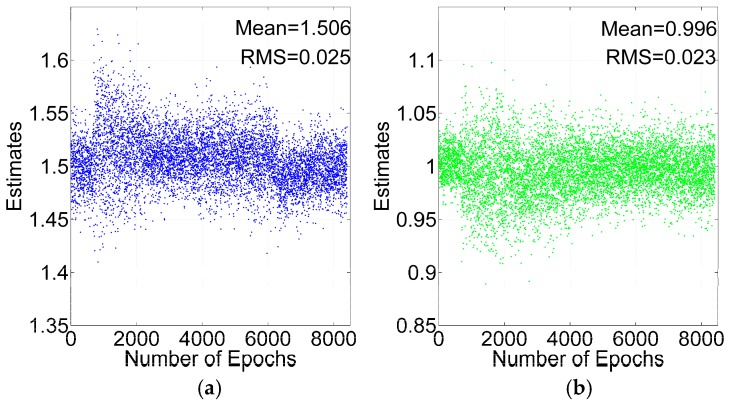
Cycle slips estimates for GPS: 1.5 Cycles (**a**); 1 Cycle (**b**).

To test the cycle slip repair method, two GPS satellites are added with one and 1.5 cycle slips at every epoch. From Table 1, ILAM can detect two cycle slips, if the number of satellites is greater than seven. Because the cycle slips should be detected before analyzing the cycle slip repair method, the condition of seven satellites with GPS is ignored in the analysis. After detecting the cycle slips with the ILAM method, the float solutions of the cycle slips can be calculated by Equation (21). The float solutions of the same two cycle slips with GPS +BDS and GPS are shown in Figure 9 and Figure 10.

As shown in Figure 9 and Figure 10, the float solutions are scattered around the true value, and the mean value of the float solutions is very close to the true value, which means that the TDCP model eliminates most of the common mode errors. The Root Mean Square (RMS) values with GPS + BDS are smaller than those with GPS. The mean RMSs are 0.018 and 0.024 cycles respectively with GPS + BDS and GPS. The result demonstrates that the accuracy of float solutions with GPS + BDS improves by 25% compared with GPS.

**Figure 11:**
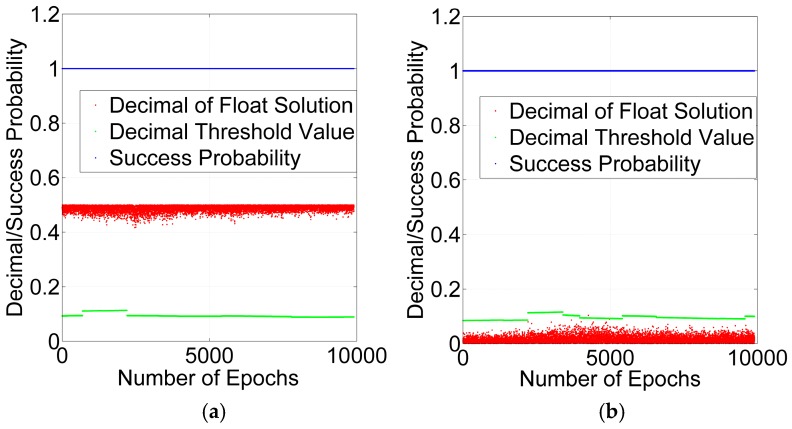
Validation by success probability and decimal test for GPS + BDS: 1.5 Cycles (**a**); 1 Cycle (**b**).

**Figure 12:**
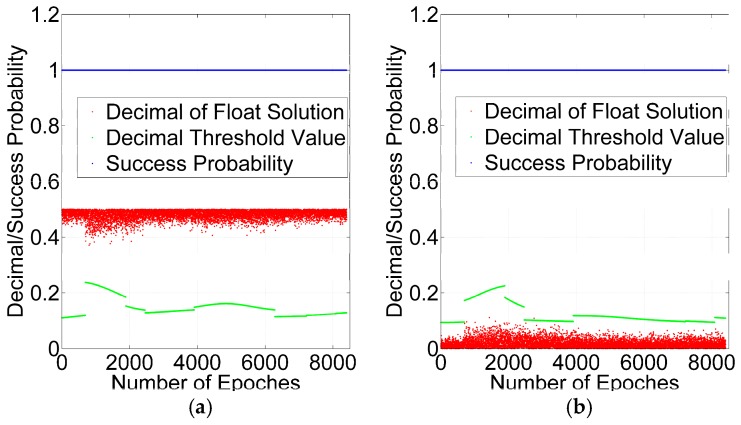
Validation by success probability and decimal test for GPS: 1.5 Cycles (**a**); 1 Cycle (**b**).

The next step is to obtain the integer cycle slips. Obviously, the one cycle slip is an integer cycle slip and should be repaired, and the 1.5 cycle slips is a non-integer outlier. The validation of the success probability and decimal test are utilized for rounding to obtain the integer cycle slips. According to Equations (22) and (23), the success probability and decimal test threshold value with GPS + BDS and GPS can be acquired at every epoch and shown in Figure 11 and Figure 12.

As shown in Figure 11 and Figure 12, the success probabilities of one cycle slip and 1.5 cycle slips are greater than 99.99%, which shows that the precision of float solutions can pass the success probability test but the non-integer outliers are not excluded by success probability. However, the decimals of the float solutions of 1.5 cycles are greater than the threshold value at all of the epochs, and the decimals of float solutions of one cycle are less than the threshold value at almost all of the epochs. The results of the GPS + BDS are similar to those of the GPS. Therefore, the decimal test can detect the non-integer outlier.

**Figure 13:**
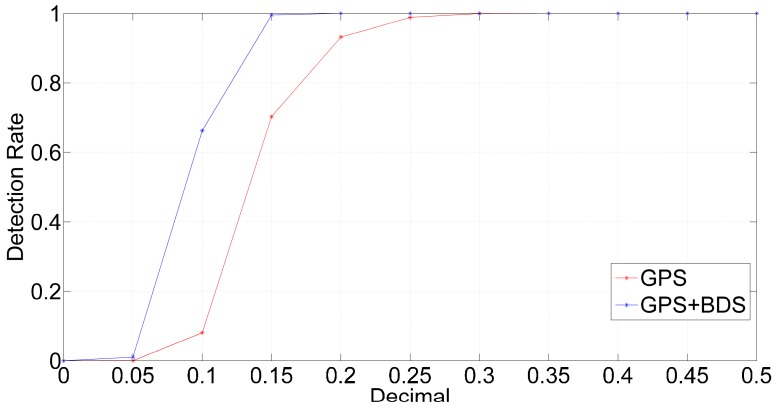
The detection rates under different decimals.

If the decimal of a non-integer outlier is greater than the threshold value, the decimal test can detect it successfully. In order to evaluate the validity of the decimal test, we make the value of 1.5 cycle slips gradually reduced from 1.5 to 1, with a decrease of 0.05 each time, and detect it at every epoch. We define the proportion of successful epochs represents the detection rate of outlier detection. Then, we can calculate the detection rates under different decimals shown in Figure 13.

As shown in Figure 13, when the decimal is 0 which means the cycle slip is an integer, the detection rates are both 0 with GPS + BDS and GPS, and the decimal test do not exclude the integer cycle slips incorrectly. When the decimal is greater than 0 and less than 0.3, the detection rates with GPS + BDS are far greater than that with GPS, and reach to 100% when the decimal is greater than 0.2. We can define the decimal when the detection rate is greater than 99.9% for the first time as the maximum decimal bias in repair. Compared to GPS, the maximum decimal bias reduces by 1/3, from 0.3 to 0.2 with GPS + BDS. Therefore, the decimal test with GPS + BDS is more effective than it is with GPS.

**Figure 14:**
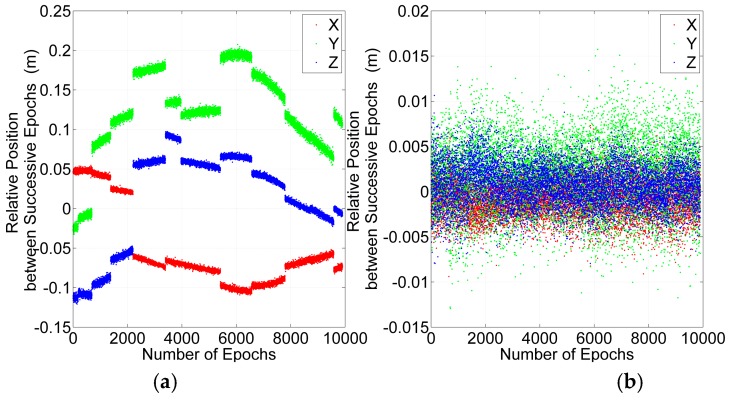
The relative position between successive epochs before (**a**) and after (**b**) cycle slips detection and repair.

The relative positions between consecutive epochs are important for velocity estimation, which is one of the major TDCP applications. It is sensitive to cycle slips and gross errors. Cycle slip detection and repair can improve the accuracy of relative positions. The relative positions are estimated by GPS + BDS before and after cycle slips detection and repair, as shown in Figure 14. Before cycle slips detection and repair, the RMSs of X, Y and Z relative position components are 0.07 m, 0.13 m and 0.06 m respectively. After that, the RMSs of X, Y and Z relative position components are 0.002 m, 0.004 m and 0.002 m respectively. The results prove that the cycle slip detection and repair is effective and important.

## 4. Conclusions

We have developed an effective geometry-based cycle slip detection and repair method with TDCP. The detection method is based on ILAM, and takes advantage of the greater number of satellites with GPS + BDS to improve the detection ability. The integer validation is utilized in repair process, including success probability and decimal test, which can detect the outliers and improve the reliability.

The experimental results demonstrate that ILAM can detect the cycle slips exactly. The detectable cycle slips number increases when there are more number of satellites with GPS + BDS, and the mean value increases by 4-fold, from two to 10, compared to GPS. In repair, the accuracy of float solutions with GPS + BDS improves by 25% compared with GPS, and the mean RMSs of float solutions are 0.018 and 0.024 cycles, respectively. With the use of the decimal test for validation, non-integer outliers can be detected, and compared to GPS, the maximum decimal bias reduce by 1/3, from 0.3 to 0.2 with GPS + BDS. All above results imply that the proposed method can take full advantage of multi-constellation. In future work, the applications such as RTK and PPP with high sampling rate are expected to use this proposed method to fix cycle slips without limitations on the number of frequencies.

## Figures and Tables

**Table 1:** The Detectable Cycle Slips Number for Different Number of Satellites.

System	GPS + BDS						GPS			
Number of Satellites	16	17	18	19	20	21	7	8	9	10
Detectable Cycle Slips Number	9	9	10	11	11	12	1	2	3	4
